# The role of LEAP2 on cognitive impulsivity after refeeding: evidence from a preclinical study in female mice and from patients with anorexia nervosa

**DOI:** 10.1038/s41398-026-03912-y

**Published:** 2026-03-05

**Authors:** Chloé Tezenas du Montcel, Héloïse Hamelin, Nicolas Lebrun, Philibert Duriez, Nicolas Ramoz, Philip Gorwood, Odile Viltart, Virginie Tolle

**Affiliations:** 1 Université Paris Cité, Institute of Psychiatry and Neuroscience of Paris (IPNP), INSERM U1266, Team “Vulnerability to Psychiatric and Addictive Disorders”, 75014 Paris, France; 2 GHU-Paris Psychiatrie et Neurosciences, Hôpital Sainte-Anne (CMME), F-, 75014 Paris, France; 3 Université de Lille, SCALab - Sciences Cognitives et Sciences Affectives, CNRS UMR 9193, PsySEF Faculty, F-59653 Villeneuve d’Ascq, France; 4 Present Address: Metabolism and Nutrition Research Group, Louvain Drug Research Institute, UCLouvain, Belgium Brussels,; 5 Present Address: McGill University, Mental Health Institute of the Douglas Hospital, H4H1R3 Montreal, QC Canada

**Keywords:** Psychiatric disorders, Neuroscience

## Abstract

Recents findings suggest that the ghrelin/LEAP2 (Liver Expressed Antimicrobial Peptide 2) ratio impacts the dynamics of reward sensitivity, and that LEAP2 may serve as a biomarker of remission in patients with anorexia nervosa (AN). We hypothesized that the ghrelin/LEAP2 ratio influences impulsive food choices following chronic food restriction and refeeding. Impulse control and plasma ghrelin and LEAP2 concentrations were evaluated in a longitudinal study of 30 female patients with AN after weight restoration and 6-months following discharge to evaluate their weight gain status. Cognitive impulsivity was also assessed in young C57Bl6/J female mice at baseline, after 15 days of 50% quantitative food restriction and after 10 days of refeeding. We collected blood for ghrelin and LEAP2 measurement and brain structures involved in metabolic response, reward and cognitive control. The ghrelin/LEAP2 ratio was negatively correlated with impulse control in patients after weight restoration, but only in those who maintained stable weight gain after discharge. In mice, food restriction increased cognitive impulsivity and refeeding only partially restored this phenotype compared to control conditions. Cognitive impulsivity was also positively correlated with plasma LEAP2 levels but not with the expression of the key hypothalamic neuropeptides or mesocorticolimbic dopamine DRD1/DRD2 receptors. Our results suggest that the interaction between LEAP2 and cognitive impulsivity is affected by changes in nutritional status in patients and female mice. Metabolic and cognitive consequences of food restriction may influence how food choices are modified in patients with AN and may be associated with a greater likelihood of achieving stable weight gain.

**Figure:**
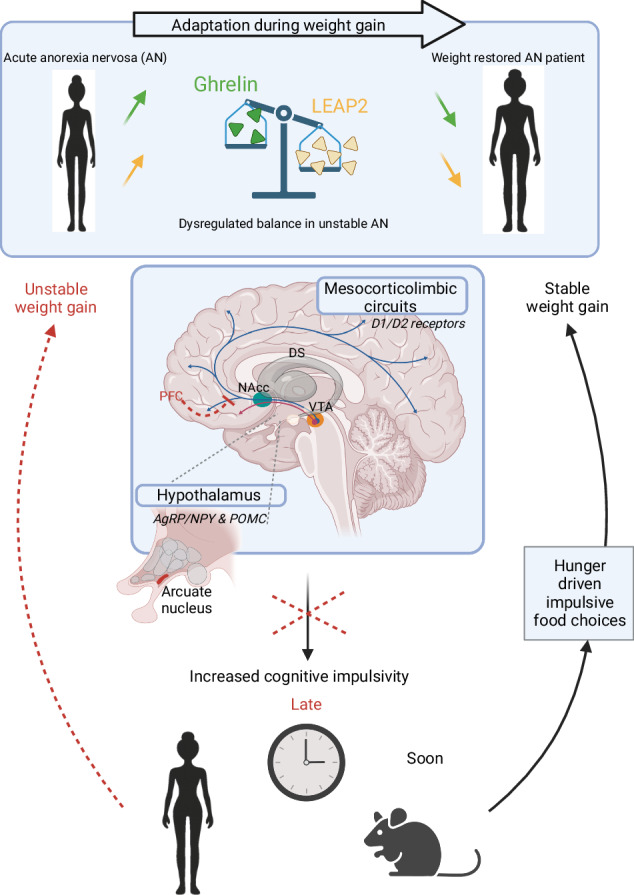
Schematic representation of the current findings and hypothesis regarding the role of the ghrelin/LEAP2 ratio on the modulation of cognitive impulsivity following refeeding and its effects on weight gain stability in AN.

Schematic representation of the current findings and hypothesis regarding the role of the ghrelin/LEAP2 ratio on the modulation of cognitive impulsivity following refeeding and its effects on weight gain stability in AN.

## Introduction

Anorexia nervosa (AN) is a severe psychiatric disorder characterized by chronic food restriction and excessive physical activity resulting in severe weight loss. Although current treatment strategies focusing on weight restoration are beneficial to patients, between 25 and 38% of patients with AN relapse within the year following hospital discharge according to a large meta-analysis [1]. There is an urgent need to identify biomarkers to enable the development of targeted interventions for patients at high risk of relapse. A recent study showed that, among the neuropsychological features identified in patients with AN, there is an increase in cognitive instability, which is often associated with impaired impulse regulation [2]. However, low cognitive impulsivity appears to be specifically associated with restrictive subtypes and positively associated with the severity of anorexic symptoms [3, 4]. Cognitive impulsivity can be defined as the inability to inhibit motivational or emotionally charged processes. It is commonly assessed using the Delay Discounting Task (DDT), which evaluates an individual’s tendency to prioritize smaller immediate over larger delayed rewards. Delay discounting is calculated as the rate at which the value of the delayed option decreases over time. Steeper discounting reflects increased cognitive impulsivity (*i.e*. the preference for immediate options) [5]. Patients diagnosed with AN have been shown to favor delayed options, a preference that aligns with lower delay discounting (*i.e*. lower impulsivity) [4]. Some clinicians support the hypothesis that delay discounting should be regarded as a stable personality trait [6–9]. Indeed, decreased delay discounting has been reported in recovered patients compared to healthy controls [10]. In contrast, others have shown that weight restoration in AN is associated with an increased preference for delayed gratification compared to the undernourished state [4, 11–13]. These findings suggest that the feeding status may influence delay discounting in patients with AN. These inconsistencies in the literature underscore the need for studies that specifically address the role of metabolic sensors in cognitive impulsivity. Although the etiology of AN remains largely unknown, it is now considered as a disorder with both metabolic and psychiatric origins. Metabolic sensors are thought to play a critical role in the vicious cycle of behaviors leading to compulsive food self-restriction and weight loss [14, 15]. Recent studies have indicated a correlation between metabolic and nutritional status and cognitive impulsivity. Bernardoni et al. observed a negative correlation between cognitive impulsivity and plasma concentrations of the desacyl form of ghrelin in patients with acute AN but not in recovered patients or in healthy controls [16]. Ghrelin, an orexigenic peptide that increases with energy deprivation, has also been identified as a pro-impulsive hormone, both in preclinical models and healthy individuals, the latter exhibiting increased cognitive impulsivity after fasting [17–20].

LEAP2 (Liver Expressed Antimicrobial Peptide 2) is an endogenous antagonist of ghrelin, that counteracts ghrelin’s orexigenic and potentially pro-impulsive actions [21, 22]. We previously demonstrated that plasma LEAP2 levels decreased, while ghrelin levels and the ghrelin/LEAP2 ratio increased following food restriction or fasting in mice and humans [23]. Refeeding has been associated with increased plasma LEAP2 and decreased plasma ghrelin levels and the ghrelin/LEAP2 ratio. In contrast to those findings in physiological conditions, our longitudinal follow-up study of patients with acute AN revealed that plasma LEAP2 levels were higher in undernourished patients than in those who had been refed, particularly in patients who relapsed within 6 months following hospital discharge [23]. These results suggest that the way these metabolic sensors adapt to chronic food restriction differs in AN compared to healthy conditions. Abnormal LEAP2 regulation could serve as a promising biomarker of relapse in AN. Recent studies have also linked LEAP2 with measures of impulsivity in human subjects, abnormal LEAP2 regulation in AN may be associated with cognitive impulsivity and the risk of unstable remission after weight restoration [18, 24, 25].

Ghrelin’s pro-feeding effects are primarily relayed in the arcuate nucleus (ArcN) of the hypothalamus through a direct activation of the orexigenic NPY/AgRP neurons and an indirect action via GABAergic inhibition of anorexigenic proopiomelanocortin (POMC) neurons [26]. In contrast, LEAP2 inhibits the activation of NPY/AgRP neurons in the ArcN and activates POMC neurons [27–29]. The sensitivity to delayed gratification implicates the following neural substrates: the dorsal striatum (DS) and the nucleus accumbens (NAc), which play a role in reward sensitivity and valuation and the prefrontal cortex (PFC) which is involved in decision-making and cognitive control [30]. It has been demonstrated that both ghrelin and LEAP2 modulate dopamine neurons within the mesocorticolimbic circuit [18, 31]. Consequently, these metabolic sensors may play a pivotal role in delay discounting by modulating dopaminergic transmission in these structures. Specifically, we hypothesized that LEAP2 could suppress ghrelin’s actions by inhibiting mesocorticolimbic circuits. This would result in low cognitive impulsivity, as evidenced by low delay discounting (*i.e*. a preference for delayed rewards in the DDT task), which is observed in patients with acute AN. Therefore, we predict that plasma LEAP2 levels would be associated with changes in delay discounting during refeeding following food restriction by modulating the circuits that regulate cognitive impulsivity.

To this end, we investigated the potential relationship between LEAP2 and cognitive impulsivity following refeeding in patients with AN, as well as in a preclinical study involving female mice. First, we examined whether the relationship between impulse control and LEAP2 plasma levels contributes to relapse risk in patients with AN after weight restoration. Next, we examined the relationship between ghrelin, LEAP2 concentrations, and cognitive impulsivity in a preclinical model mimicking the metabolic state of patients with AN before and after weight restoration. This model involved exposing young female mice to prolonged food restriction, followed by refeeding. We also assessed the expression of hypothalamic neuropeptides influenced by undernutrition and refeeding states as well as dopamine DRD1/DRD2 receptors expression in mesocorticolimbic structures.

## Materials / subjects and methods

### Clinical study (Study 1)

This study is part of an ongoing longitudinal study devoted to exploring the remission process in AN (Clinical trial Ref. NCT04560517) validated by the Ethical Committee (CPP 19.07.26.54412). The protocol has been described in our previous related publication [23]. Thirty female patients aged 18 to 60 years old diagnosed with AN according to the DSM 5 criteria were included in a 4-months multidisciplinary refeeding program in a department specialized in eating disorders after obtention of written informed consent. Following refeeding, the stability of weight gain was then evaluated 6 months after discharge (and considered as “stable” if the body mass index was over 18.5 kg/m^2^ and “unstable” otherwise). Visit consisted of a clinical evaluation of body mass index (BMI), a blood sample for metabolic explorations, and a psychiatric assessment of AN subtype (Restrictive “AN-R” n = 23, or Bingeing/Purgeing “BP” n = 7) and eating disorder symptoms using the Eating Disorder Inventory (EDI-2) questionnaire [32]. EDI-2 is a self-report questionnaire that assesses various behaviors and attitudes associated with eating disorder pathology, it includes 11 subscales evaluating psychological characteristics, amongst impulse regulation that reflects tendency to impulsivity. All patients gave informed consent. Blood was collected after an overnight fast, centrifuged at 4 °C and plasma was acidified with HCl at a final concentration of 0.1 N. Samples were stored at −80 °C and assayed within 6 months using commercial EIA assay kits (Ref. A05106 for human ghrelin, Bertin Bioreagents, Montigny le Bretonneux, France and Ref. EK-075-40 for human LEAP2, Phoenix Pharmaceuticals, Burlingame, USA) (See Supplemental material for more detailed information).

### Preclinical study (Study 2)

#### Animals

Seven-week-old C57Bl6/J female mice (Charles River Laboratories, L’Arbresle, France) were group housed (2–5 animals/cage) in Plexiglas cages (L18 x l36 x H12 cm) in a specific pathogen-free environment with a temperature between 20 and 22^◦^C, on a 12 h/12 h light-dark cycle. Baseline body weight and *ad libitum* food intake were measured after one week of habituation to the cage environment. The mean *ad libitum* food intake was calculated as the quantity of food consumed per cage. Caloric restriction was then calculated for each cage as 50% of the cage *ad libitum* food intake. Food was distributed as individual pellets in the cage and individual food restriction was controlled by measuring each mouse’s body weight. Mice had free access to standard chow diet (3% fat, 16% protein, 60% carbohydrate, 4% fibers, 2.79 kcal/g; Safe A04). All experiments were carried out according to the European Communities Council Directives (86/609/EEC) and approved by Regional Ethics Committee (CEEA.34) of the University Paris Cité.

#### Experimental paradigms

To evaluate the impact of food restriction on cognitive impulsivity, we used a progressive food restriction procedure as previously described [33]. Two experiments were performed (Fig. S2A and Supplemental material for detailed protocols). Sample size estimation was chosen, based on previous studies. In **experiment 1** (n = 8/group), mice were randomized in a control (CT) and food restricted group (FR) based on their *ad libitum* body weight after a week of habituation to the cage. For a baseline evaluation of individual cognitive impulsivity, mice were first placed under mild food restriction with a target at 85–90% of the *ad libitum* body weight to enhance motivation for reward (DDT1). Then to assess the impact of food restriction, FR mice were exposed to a 15-day 50% food restriction while CT mice were submitted to similar mild food restriction as in DDT1 and cognitive impulsivity was assessed again during DDT2. All animals were sacrificed at the end of the protocol to collect blood. One mouse per group had to be excluded because it did not reach body weight criteria. In **experiment 2** (n = 8–12/group), a group of food restricted + refeeding (FR + R) was added and animals were again randomized based on body weight after a week of habituation. After DDT1, both FR and FR + R mice were exposed to the food restriction protocol then performed DDT2. Mice of the FR group were then sacrificed and FR + R mice followed 10 days of *ad libitum* refeeding before DDT3. Animals of CT and FR + R groups were sacrificed for brain and blood samples at the end of the protocol.

#### Operant conditioning paradigm

##### Delay-discounting task (DDT)

**Fig. 1:**
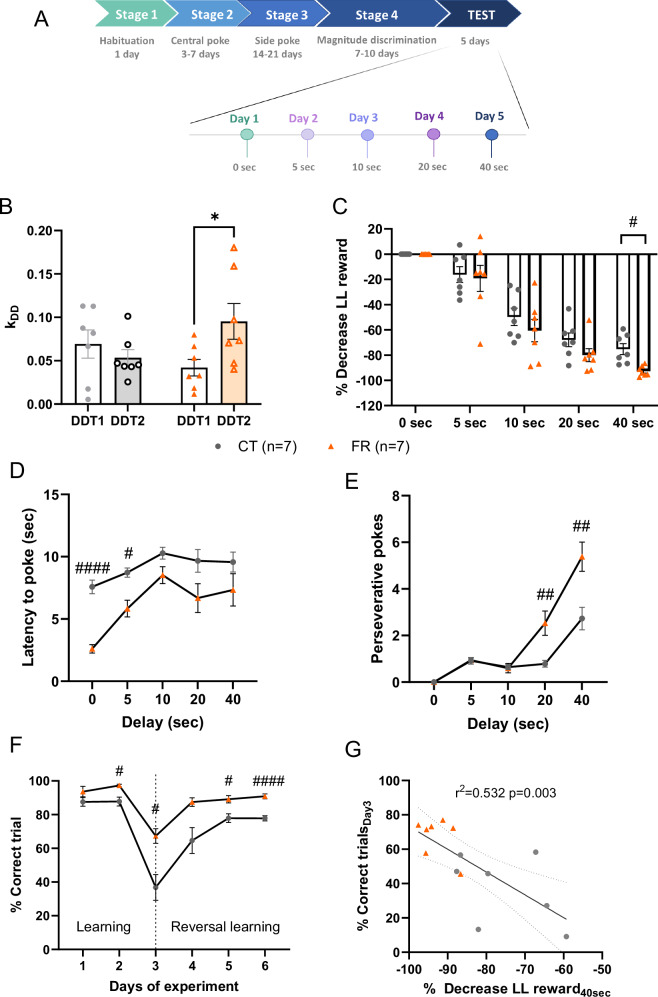
Description of the training and the DDT stages and behavioral exploration in food restricted mice. **A** Schematic representation of training and testing sessions in the DDT paradigm. **B** Devaluation coefficient (k_DD_) based on a hyperbolic model in FR compared to CT mice. **C** Preference for the larger reward with increasing delays (5–40 sec) in FR compared to CT mice. **D** Motor impulsivity evaluated with the latency to poke for the LL reward in FR compared to CT mice. **E** Number of perseverative pokes in the LL side in FR compared to CT mice. **F** Number of correct trials during the reversal learning stage in FR compared to CT mice. **G** Simple linear correlation between the percentage of correct trials on day 3 of the RL task and the percentage decreased choice for the LL reward on the 40-sec delay in DDT2. Data are expressed as mean ± sem. Within group comparison: *p < 0.05. Between groups comparison: #p < 0.05, ##p < 0.01, ####p < 0.0001. RM: repeated measures, RL: Reversal Learning, n = 7 mice per group. DDT Delay Discounting Task, CT Control, FR Food Restriction, k_DD_ Devaluation coefficient, LL Large Late (delayed gratification).

We designed a delay-discounting task adapted from a previous protocol [34] (Fig. S2B, See Supplemental material for details). The animals performed a daily 40 min session in an operant conditioning paradigm involving successive trials. The protocol was divided into 4 training stages and the DDT test (Fig. 1A). Animals were first trained to activate side pokes to get a liquid reward (Stages 1 to 3, Fig. 1A). In the last training stage (Stage 4), animals had to discriminate that one side was associated with a small (20 µL) reward and the other with a large (60 µL) reward. Magnitude discrimination was confirmed when 80% of choices were made for the large reward in two successive sessions with an inter-session variance under 10%. During the test, the large reward was delivered with an increasing delay each day (0 sec, 5 sec, 10 sec, 20 sec, 40 sec) while the small reward was delivered immediately (Fig. 1A). Animals had to choose between a “Small Soon” reward (SS) and a “Large Late” reward (LL) as represented in Fig. S2B. We recorded the number of correct and incorrect trials (respectively LL or SS), perseverative pokes (pokes during delay) and latency to poke. Preference for the LL option was calculated as the percentage of LL choice in completed trial during the session for each delay (% LL/LL + SS). To integrate the interindividual differences on the magnitude discrimination, the decrease in preference for the LL option was calculated as the difference between the preference for the LL option when delay (x) was applied and the baseline preference for the LL option when delay (x) was not applied (delay 0).

$$\% \,\mathrm{decrease}\,\mathrm{LL}\,\mathrm{choice}=100* \frac{\mathrm{preference}\,\mathrm{for}\,\mathrm{LL}\left(\mathrm{delay}x\right)-\mathrm{preference}\,\mathrm{for}\,\mathrm{LL}(\mathrm{delay}0)}{\mathrm{preference}\,\mathrm{for}\,\mathrm{LL}\left(\mathrm{delay}0\right)}.$$

Delay discounting was estimated with a discounting parameter (k_DD_) as the rate at which the subjective value of the reward decreases with larger delays (higher KDD scores were interpreted as increased cognitive impulsivity). We calculated k_DD_ using a hyperbolic model, recognized as the most reliable criteria to interpret DDT in humans [34, 35], and calculating the best-fit value in a non-linear curve fit model applied to the % decrease LL choice as a function of delay curve. We used a nonlinear regression equation function on GraphPad Prism 10.0 (Abacus Concept, Berkeley, CA, USA) implemented with our hyperbolic model described as*:*

$${\rm{kDD}}=\frac{\frac{100}{ \% {\rm{decrease\; LL\; choice}}}-1}{{delay}(\sec )}$$

A single k_DD_ value was obtained for each animal at each DDT stage. Motor impulsivity was evaluated through the number of perseverative pokes during the delay and the latency to poke for the large or the small reward, expressed in seconds.

##### Reversal learning task

To ensure that the changes observed in the delay discounting were not due to altered cognitive flexibility and that our results were not biased by impaired flexibility to food cues in response to food restriction, we tested cognitive flexibility in experiment 1 using a reversal learning task. After the DDT, animals were exposed to a Fixed-ratio in which only the LL side remained rewarded with the delivery of 40 µL of liquid reward. Mice had to reach a criterion of 75% of successful trials for two consecutive sessions before moving to the reversal trial. For the reversal trial, the rewarded hole and the non-rewarded hole were reversed. The percentage of correct trials was calculated as the number of rewarded pokes on total number of pokes.

#### Brain tissue and blood samples collection

For brain microstructure collection, brains were withdrawn and placed on ice into an acrylic mouse brain matrix for coronal sections with 1 mm spacing (Ref. 15050, Ted Pellan Inc., CA, USA). Coronal sections containing the prefrontal cortex (1.5–3.5 mm from bregma), striatum (1.5––0.5 from bregma) and hypothalamus (−0.5–−2.5 from bregma) were removed. The whole hypothalamus and PFC were dissected with precision scissors and the dorsal and ventral striatum (including the nucleus accumbens) were collected using a 1 mm diameter micropunch according to the mouse brain atlas [36] and immediately flash frozen in liquid nitrogen.

Blood was collected at sacrifice from trunk blood on an EDTA-coated tube supplemented with PHMB (p-Hydroxymercuribenzoic acid), a cysteine protease inhibitor, at 0.4 mM final concentration in blood. Samples were centrifuged to collect plasma. Plasma was aliquoted in 2 tubes, one aliquot was immediately acidified with HCl (final concentration of 0.1 N) to preserve ghrelin acylation, then stored at −80 °C until assays. Plasma concentrations of ghrelin and LEAP2 were evaluated with commercial EIA kits (Ref. A05117 for mouse/rat ghrelin, Bertin Bioreagents, Montigny le Bretonneux, France and Ref. EK-075-40 for LEAP2, Phoenix Pharmaceuticals, Burlingame, USA) as previously performed (See Supplemental material).

#### RT-qPCR

Total RNA was extracted and cDNA was obtained from reverse transcription of 1 μg of total RNA. Quantitative real-time PCR was performed using SYBR Green technology (LightCycler® 480 SYBR Green I Master (Roche Diagnostics, Meylan, France) or PowerTrack SYBR Green (Applied Biosystems, Foster City, CA, USA) on the LightCycler 480 system (Roche Diagnostics, Meylan, France). Target genes were Agouti-Related Protein (*AgRP*), Neuropeptide Y (*NPY*), Proopiomelanocortin (*POMC*), Growth Hormone Secretagogue Receptor (*GHSR*), Leptin receptor (*LepR*) as well as dopamine receptors *DRD1* and *DRD2* (See Supplemental material for details).

### Statistical analysis

Data are expressed as mean ± SEM except if otherwise specified. After validating the normal distribution of data, we used Student t-test (two-tailed) or one way, two-way or repeated measures ANOVA followed by Sidak’s or Tukey’s post-hoc test when ANOVA were significant (p < 0.05). Non-parametric tests were applied otherwise. Pearson correlations and linear regressions were performed when data were following a normal distribution or Spearman correlations otherwise. Partial correlation explored the relationship between k_DD_, LEAP2 and the expression of DRD1 in the PFC. All statistical analysis and figures were made using GraphPad Prism 10.0 (Abacus Concept, Berkeley, CA, USA) except partial correlations that were performed on StataNow/SE 18.5. All analyses were performed in a blinded manner.

## Results

### The ghrelin/LEAP2 ratio negatively correlates with cognitive impulsivity after weight restoration in patients with AN and predicts stable weight gain (Study 1)

First, we tested which of the EDI-2 dimensions was most correlated with ghrelin, LEAP2 and the ghrelin/LEAP2 molar ratio in patients after weight restoration. Of the 11 dimensions, none of the subscales were correlated with the ghrelin/LEAP2 molar ratio in all 30 refed patients (Table 1 and Fig. S1). To distinguish between stable and unstable weight gain, as a marker of metabolic remission, we focused our analyses on patients with unstable or stable weight gain during follow-up. We found no significant correlation between the ghrelin/LEAP2 ratio and impulse regulation in patients with unstable weight gain. However, for patients with stable weight gain, we found a significant negative correlation between impulse regulation (*i.e*. cognitive impulsivity) and the ghrelin/LEAP2 molar ratio (r = −0.573, p = 0.035) (Table 1). Therefore, a lower impulse regulation subscore was associated with a higher ghrelin/LEAP2 molar ratio six months after discharge only in patients with stable weight gain. There were no statistically significant differences in impulse regulation scores between patients with stable *versus* unstable weight gain (Table S1).

**Table 1:** Correlation between the ghrelin/LEAP2 ratio and EDI-2 (total score and subscores) after weight restoration in patients with AN.

Correlation with ghrelin/LEAP2 molar ratio	All (n = 30)		Stable weight gain (n = 14)		Unstable weight gain (n = 16)	
	Spearman’s r	*p-value*	Spearman’s r	*p-value*	Spearman’s r	*p-value*
Age	0.149	0.433	−0.146	0.616	0.269	0.313
Body mass index	−0.263	0.160	0.001	0.999	−0.330	0.210
EDI-2 total	−0.172	0.364	−0.271	0.346	−0.109	0.686
Drive for thinness	−0.067	0.724	−0.347	0.223	0.178	0.505
Bulimia	0.001	0.998	−0.066	0.822	0.252	0.500
Body dissatisfaction	0.057	0.766	−0.049	0.869	0.186	0.488
Ineffectiveness	−0.024	0.898	−0.046	0.875	0.056	0.836
Perfectionnism	−0.256	0.172	−0.137	0.639	−0.341	0.195
Interpersonal Distrust	−0.027	0.887	−0.059	0.840	−0.076	0.779
Interoceptive awarness	−0.267	0.154	−0.301	0.293	−0.279	0.292
Maturity fear	−0.202	0.285	−0.460	0.099	−0.029	0.915
Ascitism	−0.164	0.387	−0.207	0.473	−0.126	0.643
Impulse regulation	−0.353	0.056	**−0.573**	**0.035**	−0.134	0.618
Social insecurity	−0.160	0.397	−0.161	0.579	−0.096	0.722

30 female patients were evaluated after weight restoration based on their remission status (stable or unstable weight gain) 6 months after discharge. Data are expressed as Spearman’s r coefficient and p-value. EDI-2: Eating Disorder Inventory-2. Significant correlations are indicated in bold.

### Food restriction increases cognitive impulsivity in mice (Study 2, experiment 1)

First, we investigated the consequences of prolonged food restriction on delay discounting (*i.e*. the preference for immediate rewards) that evaluates cognitive impulsivity in female mice exposed to 15 days of food restriction, which led to a 25% *ad libitum* body weight loss (Fig. S2B). Food restriction increased delay discounting as evaluated by a higher discounting parameter k_DD_ in DDT2 compared to DDT1 (p = 0.029 DDT2 *vs* DDT1) (Fig. 1B) as well as a lower preference for the LL reward at the longest delay of 40 sec (p = 0.012 DDT2 *vs* DDT1) (Fig. 1C) in experiment 1. Food restriction had similar consequences on motor impulsivity: there was a decrease in the latency to poke in FR compared to CT conditions (Delays 0 and 5 sec, p < 0.0001 and p = 0.0203 CT *vs* FR, respectively) (Fig. 1D) and an increased number of perseverative pokes (Delays 20 and 40 sec, p = 0.013 and p = 0.043 CT *vs* FR, respectively) (Fig. 1E). To ensure that the increased delay discounting in food restricted mice was not due to altered cognitive flexibility, we performed a reversal learning task. FR mice exhibited a slightly higher percentage of correct trials on the last day of learning (p = 0.016 CT *vs* FR) and a higher percentage of correct trials from the first session of reversal learning (Day 3, p = 0.011 CT *vs* FR) until the end of the test compared to CT mice (Fig. 1F). The percentage of correct trials on day 3 was negatively correlated with the percentage of decrease in the LL reward (r = −0729, p = 0.003) (Fig. 1G). We found no significant correlation between k_DD_ and plasma concentrations of ghrelin, LEAP2 or the ghrelin/LEAP2 molar ratio in either control or food restricted conditions (Table S2).

### Refeeding partially restores food restriction-induced heightened cognitive impulsivity (Study 2, experiment 2)

**Fig. 2:**
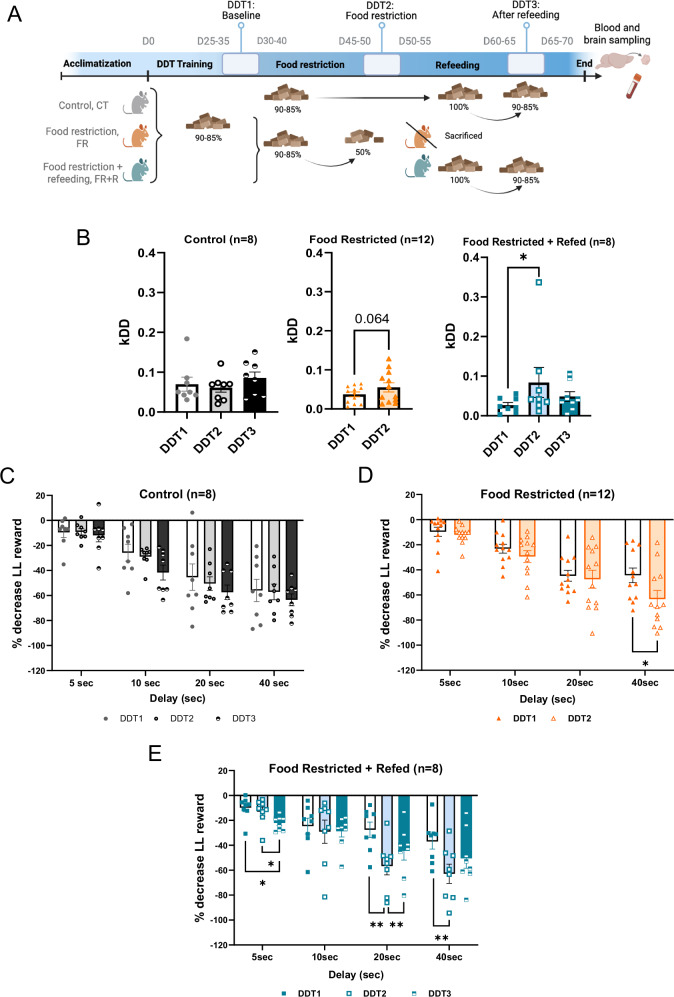
Refeeding partially restores food restriction-induced enhanced cognitive impulsivity in female mice. **A** Timeline of the experiment and representation of the three experimental groups (designed with Biorender). **B** Comparison of the devaluation coefficient (k_DD_) in the FR and FR + R groups. **C**–**E** Percentage of decrease in the LL reward in CT (**C**), FR (**D**) and FR + R (**E**) groups. Data are expressed as mean ± sem and individual values. *p < 0.05, **p < 0.01. LL Large Late, SS Small Soon, k_DD_ devaluation coefficient.

**Fig. 3:**
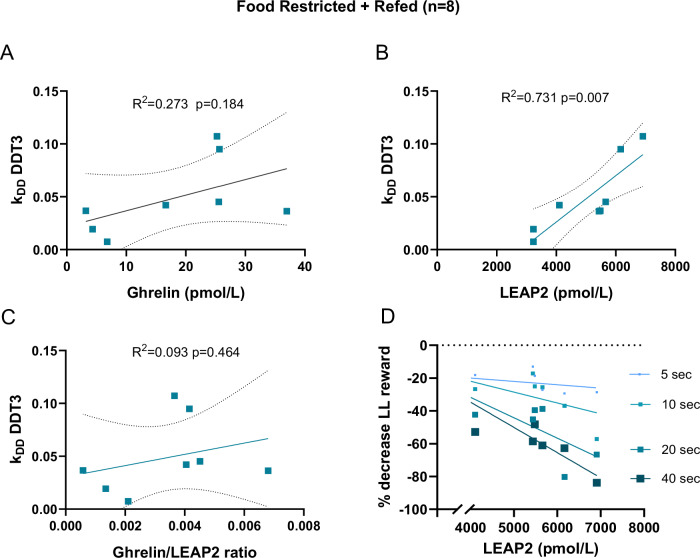
Correlation between the devaluation coefficient k_DD_ during DDT3 and plasma levels of ghrelin, LEAP2 and the ghrelin/LEAP2 ratio in FR + R conditions in female mice. **A-C** Simple linear regressions between the devaluation coefficient (k_DD_) on DDT3 and plasma levels of ghrelin (**A**), LEAP2 (**B**), and the ghrelin/LEAP2 molar ratio (**C**) in refed animals. **D** Simple linear regression between plasma levels of LEAP2 and the percentage decrease in preference for the LL reward on DDT3. Data are expressed as the coefficient of determination (r^2^) and p-value. Dotted lines represent the 95% confidence band of the best fit line. CT control, DDT delay discounting task, LL Large Late, FR food restriction, FR + R food restriction + refeeding, LEAP2 Liver Expressed Antimicrobial Peptide 2, k_DD_ Coefficient of devaluation. Correlations were performed using simple (**A**–**C**) or multiple (**D**) linear regression.

To examine the effects of food restriction on cognitive impulsivity, mice were exposed to 15 days of caloric restriction, after which they were fully refed, as described in Fig. 2A. Body weight decreased significantly during DDT2 in the FR and FR + R groups and was fully restored with refeeding (Fig. S2C). We further validated that the altered mRNA expression of main hypothalamic biomarkers of energy status, which was induced by food restriction (FR) (p < 0.0001 CT *vs* FR), was reversed by refeeding (p < 0.0001 FR + R *vs* FR) and completely normalized to CT mice (Fig. S3A). We found no differences either in plasma levels of ghrelin, LEAP2 or the ghrelin/LEAP2 molar ratio between refed and control animals (data not shown). Next, we explored the variation of the devaluation coefficient (k_DD_) and the percentage decrease in LL reward (Fig. S3A). Delay discounting (*i.e*. the preference for immediate rewards) remained stable across tests in the CT group (Fig. 2B, C) and increased during DDT2 in the FR (k_DD_ DDT1 *vs* DDT2 p = 0.064) and FR + R groups (k_DD_ DDT1 *vs* DDT2 p = 0.026) (Fig. 2B, D, E). The percentage decrease in LL reward with food restriction in the FR + R group was significant for the 20 and 40 sec delays (DDT1 *vs* DDT2, p = 0.008 and p = 0.003 for 20 and 40 sec, respectively) (Fig. 2E). Refeeding decreased k_DD_ in the FR + R group (Fig. 2B) and preference for the LL reward at longer delays (Fig. 2E), though neither change reached statistical significance. Interestingly, refed animals exhibited a decreased preference for the LL reward compared to the baseline state (DDT1 *vs* DDT3 p = 0.049) and food restricted conditions (DDT2 *vs* DDT3 p = 0.018) at the 5-sec delay (Fig. 2E). Therefore, refeeding partially reversed food restriction-induced cognitive impulsivity for the longer delays, and increased cognitive impulsivity for the shorter ones. We also found a significant positive correlation between plasma LEAP2 concentrations and k_DD_ in refed animals only (r = 0.856, p = 0.007) but no correlation between ghrelin or the ghrelin/LEAP2 ratio and k_DD_ in any of the experimental conditions (Table S3 and Fig. 3A–C). Higher plasma LEAP2 concentrations were also positively associated with stronger reductions in LL reward preference for the longer delays (correlation between plasma LEAP2 and percentage decrease in LL reward, r = −0.811, p = 0.014 at 20 sec and r = −0.927, p = 0.001 at 40 sec) (Fig. 3D).

### The association between LEAP2 and cognitive impulsivity during refeeding cannot be explained by changes in hypothalamic neuropeptides or mesocorticolimbic dopamine receptor expression (Study 2, experiment 2)

To decipher the pathways possibly mediating LEAP2’s association with cognitive impulsivity in refed mice, we performed correlation analyses of plasma LEAP2 levels with the expression of genes encoding hypothalamic sensors of energy status and mesocorticolimbic dopaminergic DRD1/DRD2 receptors. We found no significant correlation of cognitive impulsivity (k_DD_ scores) or LEAP2 with the expression of any hypothalamic genes (AgRP, NPY, POMC or GHSR*)* while ghrelin was positively correlated with AgRP expression (r = 0.726, p = 0.042) and NPY (r = 0.751, p = 0.032). These results suggest that LEAP2’s influence on cognitive impulsivity is not mediated by the adaptive transcriptional activity of LEAP2 on its hypothalamic targets such as GHSR, NPY/AgRP and POMC neurons (Fig. S3B, Fig S4 and Table S4).

**Fig. 4:**
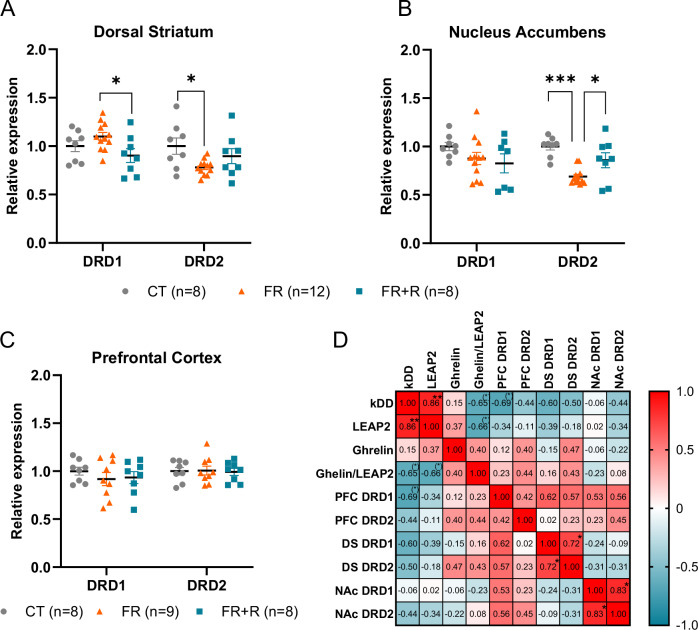
Expression of dopamine receptors is modulated by the nutritional status but is not associated with cognitive impulsivity during refeeding in female mice. **A**–**C** Expression of dopaminergic receptors DRD1 and DRD2. **D** Correlation matrix between k_DD_ and the expression of dopaminergic receptors DRD1 and DRD2 in the PFC, DS, NAcc in the FR + R group. Data are expressed as mean ± sem and individual values. *p < 0.05, ***p < 0.001. CT control, DS dorsal striatum, FR food restricted, FR + R food restricted + refed, k_DD_ devaluation coefficient, NAc nucleus accumbens, PFC prefrontal cortex.

Furthermore, the expression of DRD1 in mesocorticolimbic structures remained unchanged in food restricted mice but refeeding decreased DRD1 expression in the DS compared to food restricted mice (p = 0.039 FR *vs* FR + R) (Fig. 4A–C). DRD2 expression was decreased in the DS (p = 0.033) and in the NAc (p < 0.0001) of food restricted animals compared to the CT group (p = 0.034 FR *vs* FR + R) and was normalized with refeeding in the NAc. In refed animals, we found a positive correlation between DRD1 and DRD2 expression in the DS (r = 0.521, p = 0.043) and the NAc (r = 0.687 p = 0.023) but not in the PFC (r = 0.175 p = 0.301) (Fig. S5A-C). Among these three regions, we found no correlation between the expression of dopaminergic receptors and k_DD_, except for a tendency toward a negative correlation between k_DD_ and DRD1 expression in the PFC (r = −0.685, p = 0.061) (Fig. 4D and Table S5). We also performed a partial correlation analysis to explore the influence of DRD1 on the correlation between LEAP2 and cognitive impulsivity. The partial correlation between LEAP2 and k_DD_ was highly significant after controlling for DRD1 (r_kDD LEAP-2. *DRD1* PFC_ = 0.909 p = 0.005). Conversely, we found a negative correlation between DRD1 expression in the PFC and k_DD_ after controlling for LEAP2 (r_kDD *DRD1* PFC_._LEAP-2_ = −0.809 p = 0.027).

## Discussion

In this study, we present evidence demonstrating that the interaction between LEAP2 and cognitive impulsivity is influenced by changes in nutritional status in both female mice and patients with AN. Firstly, we showed that a higher ghrelin/LEAP2 ratio may be associated with reduced impulsivity (*i.e*. lower scores of impulse regulation) in refed patients with AN, especially those who maintained stable weight gain. Secondly, we demonstrated that food-restricted mice exhibit a decreased preference for larger delayed rewards, indicating increased cognitive impulsivity, which was only partially restored by refeeding. Finally, we found that plasma LEAP2, not ghrelin, correlated with higher k_DD_ scores (*i.e*. increased preference for immediate rewards) in refed mice, with higher LEAP2 being associated with higher impulsivity. These findings suggest a translational alignment between human and mouse data and indicate that LEAP2 could be a potential biomarker associated with cognitive symptoms of AN.

Impulsivity has been predominantly associated with bingeing/purging subtypes of eating disorders, which are characterized by emotional dysregulation, and a poorer outcome due to comorbidities such as depressive symptoms, substance abuse, and suicide [37–39]. Increased delay discounting (*i.e*. increased cognitive impulsivity) has been reported in eating disorders characterized by compulsive eating, such as binge-eating disorder, bulimia nervosa, as well as in patients with obesity. A quantitative correlation between delay discounting, symptom severity, and body mass index has been reported in the context of eating disorders [40, 41]. Interestingly, diagnostic migration is frequent within eating disorders and patients with AN often develop compulsive eating [42, 43]. Some individuals transition from one subtype (restrictive) to another (binge eating/purging) which contributes to the challenge of maintaining clinical remission [44]. In addition to the dichotomous classification of eating disorders as restrictive *versus* bingeing/purging classification of eating disorders, impulse regulation must be considered in the context of dimensional conceptualization [45]. In AN, excessive cognitive control leads to lower impulsivity (i.e. the necessity to regulate impulses) which, in turns, reduces the salience for other stimuli (*i.e*. metabolic sensing and nutritional state) [46]. Low impulse regulation could then be interpreted as heightened drive for restriction and food deprivation. Conversely, higher impulse regulation subsequent to weight restoration could enhance the immediate rewarding aspects of eating when experiencing hunger.

Indeed, the ability to resist to impulsive food choices driven by metabolic sensing requires this increased top-down cognitive control. It has been associated with dysregulated serotoninergic activity in the PFC and with low bottom up dopaminergic inputs from the striatum to the PFC [47]. This excess of cognitive control is frequently associated with lower cognitive flexibility in patients with AN [48–50] and cognitive flexibility is known to be affected by the nutritional status [51, 52]. The present study investigated the impact of food restriction in mice, demonstrating that FR mice exhibited increased impulsivity yet maintained flexibility to food cues. This suggests a lack of excess in cognitive control in FR mice, in contrast to the observed behavior in undernourished patients. Multiple studies have explored the role of the dopaminergic network as the neural substrate of decision making. Volkow et al. implicate dopamine firing in delay discounting choice in obesity and food addiction. They suggest that the choice for an immediate option relies on phasic dopamine firing, while delayed option relies on tonic dopamine firing [53]. In our study, we observed a significant decrease of the gene expression of the inhibitory dopaminergic receptors DRD2 in the DS and NAc of food-restricted mice. Moreover, we also found a higher expression of DRD1, highly implicated in phasic dopaminergic transmission, in the DS of mice exposed to food restriction compared to refed mice. We hypothesized that chronic food restriction would impact the modulation of decision making by a mechanism involving cortico-striatal dopaminergic circuits and that weight restoration would be associated with behavioral adaptations that may stem from the period of undernutrition. However, we only observed a statistical tendency toward a correlation between cognitive impulsivity and DRD1 expression in the PFC. While these findings suggest a potential association between cognitive impulsivity and reduced DRD1 receptors expression in the PFC, further exploration is required to confirm this hypothesis.

LEAP2 has been described as antagonistic to ghrelin’s actions (orexigenic and pro-impulsive). However, our study reveals that LEAP2 levels, instead, are positively correlated with greater cognitive impulsivity in refed conditions exclusively. Recent studies show a positive correlation between LEAP2 and impulsivity in participants with overweight/obesity [24] or gambling disorder [18]. Finally, higher plasma LEAP2 concentrations were associated with faster reaction times during an attentional control task in the fasted, but not the sated, condition [25]. The observed relationship between LEAP2 and impulsivity was unexpected, given that high plasma LEAP2 would block ghrelin action at the ghrelin receptor. These results suggest that the association between metabolic sensors and decision-making processes may differ depending on the nutritional status. Indeed, we could hypothesize that an episode of chronic food restriction alters the regulation between metabolic sensors and core cognitive functions. Despite the metabolic sensors returning to physiological levels after refeeding, behavioral alterations may persist. This observed discrepancy between biochemical normalization and sustained cognitive impulsivity warrants long-term neurobiological adaptations in patients with AN which may contribute to the chronicity or to the progression of the symptoms over time.

Previous studies on AN reported high plasma levels of ghrelin despite persistent food restriction in patients [54–56]. Notably, hunger has been shown to increase cognitive impulsivity in healthy controls while it does not modify cognitive impulsivity in recovered patients [12]. Our previous work also indicates that patients with acute AN exhibit abnormal LEAP2 regulation, suggesting that LEAP2 might counterbalance the orexigenic drive of ghrelin during acute stages of the disorder [21, 23]. This altered LEAP2 regulation may offer a partial explanation to the observed low cognitive impulsivity and increased ability to override the drive to eat, despite high plasma ghrelin concentrations, a situation that we do not recapitulate in mice upon chronic caloric restriction. Further studies should explore the dynamics of communication between changes in LEAP2 levels and cognitive impulsivity in patients with various nutritional statuses.

Several limitations could be made to our study. Due to the limited sample size of our clinical study, we were unable to examine the impact of AN subtype. In light of the numerous outcomes and limited number of patients, it is important that our findings be confirmed in an independent sample, with a greater number of patients evaluated prospectively. This would allow an even more robust assessment of the role of the ghrelin/LEAP2 ratio on cognitive impulsivity to predict stability of remission as a pre-hoc hypothesis. Additionally, our definition of remission relied solely on the patients’ ability *(versus* inability) to maintain the BMI acquired during the 6-months treatment process (hospitalization). It does not take into account the normalization of eating behavior or preoccupation with weight and body shape, which often take more time than normalization of body weight. In our preclinical study, a high interindividual variability in cognitive impulsivity was observed within experimental groups. To address this challenge, we opted to explore the individual’s dynamic changes between the different tests and integrated baseline performances as internal controls. Furthermore, the preclinical model used here to evaluate the impact of refeeding following food restriction did not include physical activity, which is commonly used as a core symptom in animal models of AN. While our study has shed light on the impact of the nutritional status on cognitive impulsivity in rodents, further studies should investigate the potential mitigating effect of intense physical activity on cognitive impulsivity [57]. Lastly, the present study exclusively explored transcriptional changes in refed mice, whereas changes in protein concentration or short-term alterations in dopaminergic transmission could also be involved. Recent findings have indicated the potential of LEAP2 to inhibit phasic dopamine release in the NAc in response to palatable food [31] and its capacity to decrease the GHSR’s intrinsic activity, thereby preventing its heterodimerization with DRD2 receptors [58]. In our study, we observed an absence of correlation between ghrelin levels and cognitive impulsivity suggesting a ghrelin-independent effect of LEAP2 on cognitive impulsivity. This effect may be associated with LEAP2’s inverse agonist activity on the GHSR [59]. Since we only provide correlation analyses that cannot demonstrate a direct causal mechanism concerning LEAP2 and cognitive impulsivity, further studies are necessary to investigate how LEAP2 modulates in vivo dopamine release and its consequences on cognitive impulsivity.

In summary, our study provides novel insights into the ghrelin/LEAP2 system in AN and its association with cognitive impulsivity. We demonstrate that prolonged food restriction modifies cognitive impulsivity, enhancing it even after weight regain. Cognitive impulsivity is associated with LEAP2 after refeeding in mice. Successful weight restoration in patients with AN is associated with a similar reconnection of metabolic and cognitive impulsivity, which predicts stable weight gain and may influence metabolic-driven food choices. Further studies should investigate the correlation between metabolic sensors in undernourished and refed states and cognitive impulsivity in a larger number of patients with AN to validate our findings.

## Supplementary information

Supplementary information

## Data Availability

The dataset generated and analyzed in the current study is available from the corresponding author after careful review of the request.
