# Supplementary material for: The role of LEAP2 on cognitive impulsivity after refeeding: evidence from a preclinical study in female mice and from patients with anorexia nervosa
Source: Transl Psychiatry. 2026 Mar 5;16:146. doi: 10.1038/s41398-026-03912-y (PMC12987950; doi:10.1038/s41398-026-03912-y)
Supplement: Supplementary file 1 — Supplementary information [file 41398_2026_3912_MOESM1_ESM.pdf]

Supplemental information

Supplementary Material/Subjects and Methods

1. Clinical study (Study 1)

1.1. Participants

This study is part of an ongoing longitudinal study devoted to exploring the remission process in AN. Our protocol has been described in our previous related publication[1]. Inclusion criteria were: female patients between 18 and 60 years old, with DSM-5 criteria of anorexia nervosa (AN). Exclusion criteria consisted of the inability to express informed consent, severe comorbid physical or psychiatric condition. Thirty patients were included in a department specialized in eating disorders (CMME, GHU Paris Psychiatrie et Neurosciences). All participants included had three visits i/ the first visit (V1), in an undernourished state, performed in the first week after admission of inpatients, ii/ the second visit (V2) took place after four months of intensive care and before hospital discharge when participants reach a target body mass index (BMI>19 kg/m^2) therefore being considered as in a refed state, iii/ the third visit (V3) took place six months after discharge with an evaluation of the remission status (stable *versus* unstable weight gain). Stable weight gain status consisted of a maintained weight gain 6 months after discharge (BMI>18.5 kg/m^2) whereas early weight loss characterized unstable weight gain. The present study explored behavioral and metabolic markers from the visit after weight restoration as well as the remission status. The visit consisted of a clinical evaluation which included the assessment of weight, BMI, a blood sample for metabolic explorations, and a psychiatric evaluation with assessment of AN subtype (Restrictive “AN-R”, or Binge Purge “BP”) and eating disorder symptoms with Eating Disorder Inventory, EDI-2[2]. The French version of the EDI-2 was used to assess symptoms severity and different clinical dimensions of AN: drive for thinness, bulimia, body dissatisfaction, ineffectiveness, perfectionism, interpersonal distrust, interoceptive awareness, maturity feat, ascetism, impulse regulation and

social insecurity. The impulse regulation subscale was added to the later EDI-2 version to reflect the ability to regulate impulsive behavior, especially the binge behavior.

1.2. Blood collection

Blood was collected at each visit after an overnight fast on Vacutainer tubes treated with EDTA and Aprotinin (Cat#454261, Greiner Bio One SAS, Courtaboeuf, France). After collection, blood was kept at 4°C before centrifugation within 2h (1000 g for 10 min at 4°C). Plasma was aliquoted and one aliquot was immediately acidified with HCl (final concentration of 0.1N). Samples were stored at -80°C at Centre de Ressources Biologiques (CRB) of GHU Paris Psychiatrie et Neurosciences and assayed within 6 months.

2. Preclinical study (Study 2)

2.1. Food Restriction and refeeding protocol

To evaluate the impact of chronic food restriction on cognitive impulsivity in rodents, we used a progressive food restriction procedure adapted from the Food Restriction and Wheel protocol[3]. Animals were acclimatized in the facilities for a week. Then, mice and their food intake were weighted daily. Baseline food intake per cage was calculated as a mean of daily food intake on the past 5 days and considered as *ad libitum* food intake. Body weight on that day was also considered as *ad libitum* body weight.

Two experiments were performed (Figure 1A). For both experiments, mice were randomized in different groups. There were two groups of animals in **experiment 1**: control group (CT, n=7) and food restricted group (FR, n=7); and three groups of animals in **experiment 2**: control group (CT, n=8), food restricted group (FR, n=12) and food restricted + refeeding group (FR + R, n=8). Animals were placed under mild food restriction to enhance motivation for reward and to allow the learning of the DDT task with a target at 85-90% of the *ad libitum* body weight. Food was delivered daily around 5:00 PM as individual pellets of similar weight to avoid competition between mice. The mice of the control group were submitted to the mild food restriction until the end of the protocol. Mice of the FR group were exposed to a 50% calorie

restriction of their *ad libitum* food intake for 15 days. For the **experiment 2**, mice of the FR+R group were refed with *ad libitum* access to the food during 10 days after the food restriction described above.

Mice were housed 2 to 5 per cage to limit stress for behavioral tests. All animals were exposed to a mild food restriction during 25 to 35 days of training to the delay discounting task (DDT) and the first test (DDT1) was used as a baseline evaluation of the individual discounting. Then, the FR group was submitted to 15 days of food restriction as previously described during which all animals had a session of magnitude discrimination training every three days to maintain task acquisition. After the 15 days, CT and FR group performed a second DDT test (DDT2) to assess the impact of food restriction on cognitive impulsivity. Finally, animals of the CT and FR groups have performed a reversal learning test. All animals were sacrificed at the end of the protocol to collect brain and blood samples.

2.2. Delay discounting task for rodents

2.2.1. Experiments

Experiment 1. Mice were housed two per cage to limit stress for behavioral tests. All animals were exposed to a mild food restriction until the first test (DDT1) that was used as a baseline evaluation of the individual discounting. After the 15-day food restriction, CT and FR group performed a second DDT test (DDT2) to assess the impact of food restriction on cognitive impulsivity. Finally, animals of the CT and FR groups have performed a reversal learning test. All animals were sacrificed at the end of the protocol to collect brain and blood samples.

Experiment 2. Animals were housed 4-5 per cage. The procedure was similar than experiment 1, After baseline evaluation in DDT1mice of the FR and FR+R groups were submitted first to food restriction as described above and the three groups were tested on DDT2, the mice of the FR group were then sacrificed to collect brain samples. Finally, mice of the CT and FR+R group were tested a third time (DDT3) after 10 days of refeeding (see previous section). All

animals of CT and FR+R groups were sacrificed for brain and blood samples at the end of the protocol.

2.2.2. Apparatus

Behavioral explorations took place in 8 operant chambers (MedAssociates® MED-008-CT-B3, St Albans, VT, USA) on weekdays between 09:00 AM and 12:00 AM in a quiet room. Each chamber is protected from ambient noise and light being housed in an individual cabinet that is closed during the session. The operant wall contains three head entry detectors: two side holes and a central magazine where food is delivered in a food cup (Figure 1B). Target holes and food delivery are indicated with individual light cues. Liquid reward is delivered in the food cup through silicone tubing connected to a 10 mL syringe adapted on MedAssociates (St Albans, VT, USA) syringe pumps (motor speed = 3.33 rpm). We used a liquid reward mix of 1:1 strawberry flavored milk, commercially available, and strawberry flavored water added with natural sweetener (natural strawberry flavor 4 g/l + Rebaudioside A 1,75 g/l). This mix allowed a highly hedonic reward with limited caloric intake compared to pure strawberry flavored milk. The caloric intake was 285 kcal/L. Water was withdrawn from home-cages 2 hours prior to the test to trigger motivation for liquid rewards. The DDT protocol has been designed thanks to David Fuller (engineer at K-Limbic) with the K-Limbic Software®.

2.2.3. Operant conditioning paradigm

Delay-discounting task. We designed a delay-discounting task adapted from the literature (Mitchell, 2014). The animals performed one session of 40 minutes every day. During a session, the animal had to perform several trials involving a side hole choice and consumption of the corresponding reward. Two trials were separated with a 10 sec inter-trail interval (ITI), when all lights turned off (Figure 1C). The protocol was divided into 5 stages: 4 training stages and the test (Figure 1D).

1- **Habituation**: on the first day of food restriction, mice were placed in the operant chamber with 40 µL of food reward dripping in the central magazine every 2 minutes to limit neophobia.
2- **Center nose poke learning:** animals were trained to poke in the central magazine to receive a reward of 40 µL. Only pokes during the 20 sec illumination intervals were reinforced. Success was determined if the animal could get 40 rewards per session on two successive sessions and could access the subsequent stage.
3- **Side pokes learning:** animals were then trained to activate side pokes and obtain the reward in the central magazine. Left and right pokes were active during 20 seconds, indicated with a light cue and a head poke in one of the side holes delivered a 40 µL reward in the food cup indicated by the illumination of the central magazine for a maximum of 3600 seconds before a 10 seconds ITI. This stage permitted to evaluate the lateralization bias of each animal. We determined the baseline side preference considered as the side with the maximum number of pokes per session. Success was determined if the animal could get 40 rewards per session on two successive sessions and could access the subsequent stage.
4- **Magnitude discrimination:** this stage was like the previous one except one side was rewarded with a small (20 µL) reward and the other with a large (60 µL) reward. The small reward side was the preferred side determined with the baseline side preference to limit bias. The large reward side stayed the same until the end of testing. Magnitude discrimination was determined when animals chose the large reward in more than 80% of the trials per session in two successive sessions with an inter-session variance under 10%.

After training, testing consisted of a 5-day protocol. The large reward was delivered with an increasing delay each day (0 sec, 5 sec, 10 sec, 20 sec, 40 sec) and the small reward remained delivered immediately. Animals had to choose between a “Small Soon” reward (SS) and a “Large Late” reward (LL) as represented in Figure 1C-D.

The following behavioral components were recorded:

- Completed trials: trials containing a side poke during the 20 sec active phase followed by central food retrieval in the 3600 sec active phase (correct + incorrect trials).
- Correct trials: LL choice
- Incorrect trials: SS choice
- Omitted trial: no side poke or no central magazine poke during active phases.
- Perseverative pokes: side pokes during food delivery and central magazine activity.
- Latency to poke: latency to poke in the SS or LL side poke in the 20 sec of illumination of both side-pokes.

Reversal learning task. To ensure that the changes observed in the delay discounting were not due to altered cognitive flexibility and that our results were not biased by impaired flexibility to food cues in response to food restriction, we tested cognitive flexibility in experiment 1 using a reversal learning task.

The day after the DDT test, animals were exposed to a simple fixed-ratio operant conditioning task in which the side hole associated with the large reward was rewarded with a 40 µL reward (1 poke for 1 reward) and the opposite side was not rewarded anymore. We verified that mice learned the new rules and reached the success criterion of 75% of successful trials with a poke on the rewarded side for two consecutive sessions.

After the DDT, animals were exposed to a Fixed-ratio 1 (FR1) in the same apparatus as previously. Only the LL side remained rewarded with the delivery of 40µL of milk. Mice performed one session per day. A session lasted 40 minutes or stopped after 60 successful trials. Mice has to reach a criterion of 75% of successful trials (poke in the rewarded hole) for two consecutive sessions before moving to the reversal trial. For the reversal trial, the rewarded hole and the non-rewarded hole were reversed.

Behavioral data analyses. Delay discounting is calculated as the rate at which the subjective value of the reward decreases with larger delays. Delay discounting was assessed using the

preference for LL criteria (% LL/LL+SS) for each delay during the block session. The preference for the LL option was calculated as the percentage of choice for the large option compared to the number of completed trial during the session for each delay.

We integrated the interindividual differences on the magnitude discrimination estimated as the preference for LL without delay (delay of 0 sec). We therefore calculated the percentage of decrease of the preference using the preference with the delay of 0 sec as baseline. The decrease in preference for the LL option was calculated as the difference between the preference for the LL option when delay (x) was applied and the baseline preference for the LL option when delay (x) was not applied (delay 0).

$$\%\ \text{decrease LL choice} = 100 * \frac{\text{preference for LL (delay x)} - \text{preference for LL (delay 0)}}{\text{preference for LL (delay 0)}}$$

Each delay was associated with a preference expressed as a percentage for each animal. Hyperbolic model is the most reliable criteria to interpret data from a delay discounting test [1]. The hyperbolic model widely used in the literature to analyses clinical data from DDT is [2]:

$$\mathrm{V} = \frac{\mathrm{R}}{1 + \mathrm{k} * \mathrm{D}}$$

V: subjective value of the reward, R: reward value, k: discounting parameter, D: delay

We therefore tried to apply a similar model to preclinical data to facilitate the design of translational protocols that could use similar math to calculate discounting parameter. We determined a discounting parameter (k_{DD}) for each animal, calculated from a hyperbolic model applied to the % LL choice as a function of delay curve using the following formula:

$$\%\text{decrease LL choice} = \frac{100}{1 + \text{kDD} * \text{delay (s)}}$$

We used a nonlinear regression equation function on GraphPad Prism 10.0 (Abacus Concept, Berkeley, CA, USA) implemented with our hyperbolic model described as :

$$\mathrm{kDD} = \frac{\frac{100}{\%\mathrm{Decrease\ LL\ choice}} - 1}{delay\ (\mathrm{sec}\quad)}$$

The discounting parameter (k_{DD}) was calculated as the best-fit value in a non-linear curve fit model and was determined for each animal at each DDT (DDT1 and DDT2 for experiment 1, DDT1, DDT2 and DDT3 for experiment 2).

Motor impulsivity was evaluated through the number of perseverative pokes during the delay and the latency to poke for the large or the small reward, expressed in seconds.

For the reversal learning, we calculated the percentage of correct trials as the number of rewarded pokes on total number of side pokes. The number of trials increased during the reversal learning task.

2.4 Sample collection for metabolic explorations

Blood was collected at sacrifice from trunk blood on an EDTA-coated tube supplemented with PHMB (p-hydroxymercuribenzoic acid), a cysteine protease inhibitor, at 0.4 mM final concentration in blood. Samples were kept on ice and centrifuged at 4°C (1000 g for 10 min) to collect plasma. Two aliquots of plasma were prepared: one aliquot was immediately acidified with HCl (0.1N final concentration) to preserve ghrelin stability then frozen on dry ice and the second aliquot was frozen directly without acidification. Plasma samples were then stored at -80°C until assays.

2.5 RT-qPCR analyses

Total RNA was extracted using Trizol reagent (Invitrogen Life Technologies, Thermo Fisher Scientific, Waltham, USA) and cDNA was obtained from reverse transcription of 1 μg of total RNA. A RQ1 DNase step (Promega France, Charbonnières-les-Bains) was performed on total RNA before reverse transcription with High Capacity cDNA Reverse Transcription Kit (Applied Biosystems, Foster City, CA, USA). Quantitative real-time PCR was performed using SYBR Green technology (LightCycler® 480 SYBR Green I Master (Roche Diagnostics, Meylan, France) or PowerTrack SYBR Green (Applied Biosystems, Foster City, CA, USA) on the

LightCycler 480 system (Roche Diagnostics, Meylan, France). Target genes were Agouti-related Protein (*AgRP*), Neuropeptide Y (*NPY*), Proopiomelanocortin (*POMC*), Growth Hormone Secretagogue Receptor (*GHSR*), Leptin receptor (*LepR*) as well as dopamine receptors *DRD1* and *DRD2*. The comparative ΔΔCt method, where Ct is the threshold cycle at which amplified PCR product was detected, was used to assess the relative expression of the target genes normalized to the Ppia transcript (housekeeping gene). In the FR group, one hypothalamic sample and three PFC samples had to be excluded due to poor RNA quality. All primers sequences are available upon request.

3. ELISA immunoassays

Plasma concentration of acyl ghrelin (AG) was evaluated with specific enzyme-immunoassay kits (Cat#A05106 for human, CA#A05117 for mouse/rat, Bertin Bioreagents, Montigny le Bretonneux, France). All used samples came from acidified aliquot since acidification is known to preserve ghrelin stability. External quality control of the same mice and human plasma was respectively used in all assays to ensure inter-assay stability. Intra- and inter- assay coefficients of variation were <9% and <16% respectively in humans, 7% and 8% in mice. Plasma concentrations of LEAP2 were measured with enzyme-immunoassay kit (Cat#EK-075-40, Phoenix Pharmaceuticals, Burlingame, USA). The commercial kit used here recognizes both mouse and human LEAP2, i.e. LEAP2 (38-77) (Human) / LEAP2 (37-76) (Mouse) (100% cross-reactivity). External quality control of the same human plasma was respectively used in all assays to control inter-assay variation. Intra- and inter-assay coefficient of variation were respectively <10% and <15%. Concentrations were transformed in pmol/L and the Ghrelin/LEAP2 molar ratio was calculated using molar ratio.

References

1. Tezenas du Montcel C, Duriez P, Cao J, Lebrun N, Ramoz N, Viltart O *et al.* The role of dysregulated ghrelin/LEAP-2 balance in anorexia nervosa. *iScience* 2023; 107996.

2. Garner DavidM. EDI-2: Eating Disorder Inventory-2. *Odessa Psychol Assess Ressour* 1991.

3. Méquinion M, Chauveau C, Viltart O. The use of animal models to decipher physiological and neurobiological alterations of anorexia nervosa patients. *Front Endocrinol* 2015; **6**: 68.

Supplementary Tables

Table S1. Descriptive statistics of the population of the cohort study after weight restoration in patients with AN. 30 female patients were evaluated after weight restoration based on their remission status (stable or unstable weight gain) 6 months after discharge. Data are expressed as mean ± SEM. Mann Whitney paired t-test (two-tailed) and χ^2 test, $p<0.05$ considered significant. AN: Anorexia Nervosa, AN-R: Anorexia Nervosa Restrictive-type; AN-BP: Anorexia Nervosa Bingeing/Purging-type, BMI: Body Mass Index, EDI-2: Eating Disorder Inventory 2, LEAP2: Liver Expressed Antimicrobial Peptide 2. Significant differences are indicated in bold.

Table S2. Correlations between the devaluation coefficient (k_{DD}) in DDT2, metabolic status plasma biomarkers and body weight decrease in FR conditions in female mice. Data are expressed as Pearson's r coefficient, r^2 and p-value. AL: *Ad libitum,* CT: control, FR: food restricted, k_{DD}: devaluation coefficient, LEAP2: Liver Expressed Antimicrobial Peptide 2.

Table S3. Correlations between the devaluation coefficient (k_{DD}) in DDT3, plasma ghrelin, LEAP2 and ghrelin/LEAP2 ratio and percentage of body weight decrease in FR+R conditions in female mice. Data are expressed as Pearson's r coefficient and p-value. AL: *Ad libitum,* CT: control, FR+R: food restricted + refed, k_{DD}: devaluation coefficient. Significant correlation is indicated in bold.

Table S4 (refering to Figure S3B). Correlation matrix between k_{DD}, plasma levels of LEAP2, ghrelin, and hypothalamic gene expression in FR+R conditions in female mice. AgRP: Agouti Related Protein, GHSR: Growth Hormone Secretagogue Receptor, k_{DD}: devaluation coefficient, LEAP2: Liver expressed Antimicrobial Peptide 2; NPY: Neuropeptide Y, POMC: Proopiomelanocortin. Data are expressed as Pearson's r coefficient and p-value. Significant correlations are indicated in bold.

Table S5 (refering to Fig 4). Correlation matrix between the devaluation coefficient k_{DD} and the expression of dopaminergic receptors DRD1 and DRD2 in the DS, NAc and PFC

in FR+R conditions in female mice. DS: dorsal Striatum, DRD1: Dopaminergic Receptor type 1, DRD2: Dopaminergic Receptor type 2, k_{DD}: devaluation coefficient, LEAP2: Liver expressed Antimicrobial Peptide 2, Nac: nucleus accumbens, PFC: prefrontal cortex. Data are expressed as Pearson's r coefficient and p-value. Significant correlations are indicated in bold.

**Descriptive statistics**	All (n=30)	Stable weight gain (n=14)	Unstable weight gain (n=16)	Statistical test, p-value
Age (years)	26.41±1.62	24.71±1.52	27.72±2.63	U=120 p=0.829
Subtype (AN-R/AN-BP)	25 (78%)/7(22%)	10 (71%)/4(29%)	15 (83%)/3(17%)	χ^2 =0.653 p=0.419
BMI (kg/m^2)	20.02±0.081	20.22±0.091	19.84±0.115	**U=54 p=0.014**
EDI-2 score	62.430±7.640	71.14±13.30	51.59±7.834	U=93.50 p=0.321
Impulse regulation	3.067±0.717	4.429±1.217	2.000±0.725	U=80 p=0.178
Ghrelin (pmol/L)	21.35±3.80	18.43±5.55	23.90±5.29	U=79 p=0.179
LEAP2 (pmol/L)	3160±297	3195±437	2946±395	U=117 p=0.750
Ghrelin/LEAP2 molar ratio	0.007±0.001	0.006±0.001	0.008±0.001	U=114 p=0.659

Table S1.

**Correlation with kDD in DDT2**	Group	Experiment 1 (Food restriction)	
		CT	FR
Ghrelin (pmol/L)	*r*	-0.642	0.576
	r^2	0.003	0.331
	p-value	0.697	0.176
LEAP2 (pmol/L)	*r*	-0.181	0.391
	r^2	0.413	0.153
	p-value	0.119	0.386
Ghrelin/LEAP2 molar ratio	*r*	0.169	0.175
	r^2	0.028	0.031
	p-value	0.718	0.708
% AL Body weight	*r*	-0.389	-0.343
	r^2	0.150	0.118
	p-value	0.388	0.452

Table S2.

**Correlation with kDD in DDT3**	Group	Experiment 2 (Refeeding)	
		CT	FR+R
Ghrelin (pmol/L)	*r*	-0.383	0.520
	r^2	0.146	0.271
	p-value	0.350	0.186
LEAP2 (pmol/L)	*r*	-0.312	**0.856**
	r^2	0.098	**0.733**
	p-value	0.451	**0.007**
Ghrelin/LEAP2 molar ratio	*r*	-0.296	0.302
	r^2	0.087	0.091
	p-value	0.477	0.468
% AL Body weight	*r*	-0.509	-0.436
	r2	0.259	0.189
	p-value	0.198	0.181

Table S3.

**Correlation matrix**	kDD		LEAP2		Ghrelin		Ghrelin/LEAP2 molar ratio		*AgRP*		*GHSR*		*NPY*	
	Pearson r	*p-value*	Pearson r	*p-value*	Pearson r	*p-value*	Pearson r	*p-value*	Pearson r	*p-value*	Pearson r	*p-value*	Pearson r	*p-value*
kDD														
LEAP2	**0.855**	**0.007**												
Ghrelin	0.150	0.723	0.375	0.360										
Ghrelin/LEAP2	-0.647	0.083	-0.659	0.076	0.405	0.320								
AgRP	0.061	0.885	0.127	0.765	**0.726**	**0.0415**	0.320	0.439						
GHSR	0.087	0.837	-0.147	0.729	-0.171	0.686	-0.0131	0.975	-0.074	0.862				
NPY	0.024	0.955	0.111	0.794	**0.751**	**0.032**	0.348	0.398	**0.939**	**0.001**	0.067	0.875		
POMC	-0.379	0.354	-0.283	0.497	0.595	0.119	0.630	0.094	**0.843**	**0.009**	0.117	0.783	**0.841**	**0.009**

Table S4.

.

**Correlation matrix**	kDD		LEAP2		Ghrelin		Ghrelin/LEAP2 molar ratio		DS DRD1		DS DRD2		NAc DRD1		NAc DRD2		PFC DRD1	
	Pearson r	*p-value*	Pearson r	*p-value*	Pearson r	*p-value*	Pearson r	*p-value*	Pearson r	*p-value*	Pearson r	*p-value*	Pearson r	*p-value*	Pearson r	*p-value*	Pearson r	*p-value*
kDD																		
LEAP2	**0.855**	**0.007**																
Ghrelin	0.150	0.723	0.375	0.360														
Ghrelin/LEAP2	-0.647	0.083	-0.659	0.076	0.405	0.320												
DS DRD1	-0.602	0.114	-0.391	0.338	-0.149	0.725	0.163	0.700										
DS DRD2	-0.498	0.209	-0.178	0.673	0.465	0.245	0.430	0.289	**0.722**	**0.043**								
NAc DRD1	-0.061	0.897	0.017	0.970	-0.055	0.906	-0.231	0.617	-0.239	0.606	-0.306	0.504						
NAc DRD2	-0.435	0.281	-0.342	0.406	-0.223	0.595	0.077	0.856	-0.087	0.837	-0.306	0.460	**0.829**	**0.021**				
PFC DRD1	-0.685	0.061	-0.340	0.410	0.123	0.772	0.226	0.590	0.617	0.103	0.571	0.139	0.527	0.224	0.563	0.146		
PFC DRD2	-0.436	0.280	-0.112	0.792	0.405	0.320	0.442	0.273	0.022	0.959	0.234	0.577	0.229	0.621	0.445	0.269	0.419	0.302

Table S5.

Supplementary Figures

Fig S1. Scatter plots of the correlations between the ghrelin/LEAP2 ratio and impulse control after weight restoration in patients with AN (referring to Table 1). Scatter plots of correlations between ghrelin/LEAP2 ratio and impulse regulation dimension of the EDI-2 in all patients (n=30) as well as in the subgroup of patients with stable (n=14) or unstable (n=16) weight gain 6 months after discharge. LEAP2: Liver Expressing Antimicrobial Peptide 2, EDI-2: Eating Disorder Inventory 2.

Fig S2. Schematic representation of the experiments and body weight changes during food restriction and refeeding in female mice. A Timeline of the experiments (Designed with Biorender). **B** Graphic representation of the operant conditioning chamber and example of trial (Designed with Biorender). **C-D** Percentage of *ad libitum* body weight during DDT tests in experiment 1 (C) and 2 (D). Mild food restriction leads to similar weight loss in DDT1 but FR mice exhibit decreased body weight compared to CT mice in DDT2. **D** Evolution of body weight in experiment 2 after food restriction for FR and FR+R groups in DDT2. Data are expressed as mean ± sem. Within group comparisons ****p<0.0001; Between group comparison ##p<0.01, ###p<0.001. CT: control, DDT: delay discounting task, FR: food restriction, FR+R: food restriction + refeeding, RM: repeated measures.

Fig S3. Expression of hypothalamic biomarkers of the nutritional status in CT, FR and FR+R conditions and correlation matrix in FR+R conditions in female mice. A Expression of hypothalamic AgRP, NPY, POMC, GHSR and and LepR in CT, FR and FR+R mice. **B** Correlation matrix between k_{DD}, plasma levels of LEAP2, ghrelin, ghrelin/LEAP2 ratio and gene expressions of hypothalamic genes in the FR+R group (See also Table S4 for r and p-value). Data are expressed as mean ± sem. ****p<0.0001. AgRP: Agouti Related Protein, CT: Control, FR: Food Restriction, FR+R: Food Restriction + Refeeding, GHSR: Growth Hormone Secretagogue

Receptor, k_{DD}: Devaluation coefficient, LEAP2: Liver Expressing Antimicrobial Peptide 2, LepR: Leptin receptor, NPY: Neuropeptide Y, POMC: Proopiomelanocortin.

Fig S4. Scatter plots of correlations between ghrelin and the expression of hypothalamic biomarkers in FR+R conditions in female mice (associated to Fig S2B). Simple linear regression between plasma ghrelin levels and expression of hypothalamic neuropeptides AgRP, NPY, POMC or GHSR (**A**) and between expression of AgRP and NPY, POMC and NPY and POMC and AgRP (**B**). Data are expressed as coefficient of determination (r^2) and p-value. Dotted lines represent the 95% confidence band of the best fit line. AgRP: Agouti Related Protein, GHSR: Growth Hormone Secretagogue Receptor, NPY: Neuropeptide Y, POMC: Proopiomelanocortin.

Fig S5. Correlation between the expression of DRD1 and DRD2 in brain structures of the cortico-striatal network in FR+R conditions in female mice. Simple linear regression in the dorsal striatum (**A**), nucleus accumbens (**B**) and prefrontal cortex (**C**). Data are expressed as coefficient of determination (r^2) and p-value. Dotted lines represent the 95% confidence band of the best fit line. DRD1: dopamine receptor type 1, DRD2: dopamine receptor type 2.

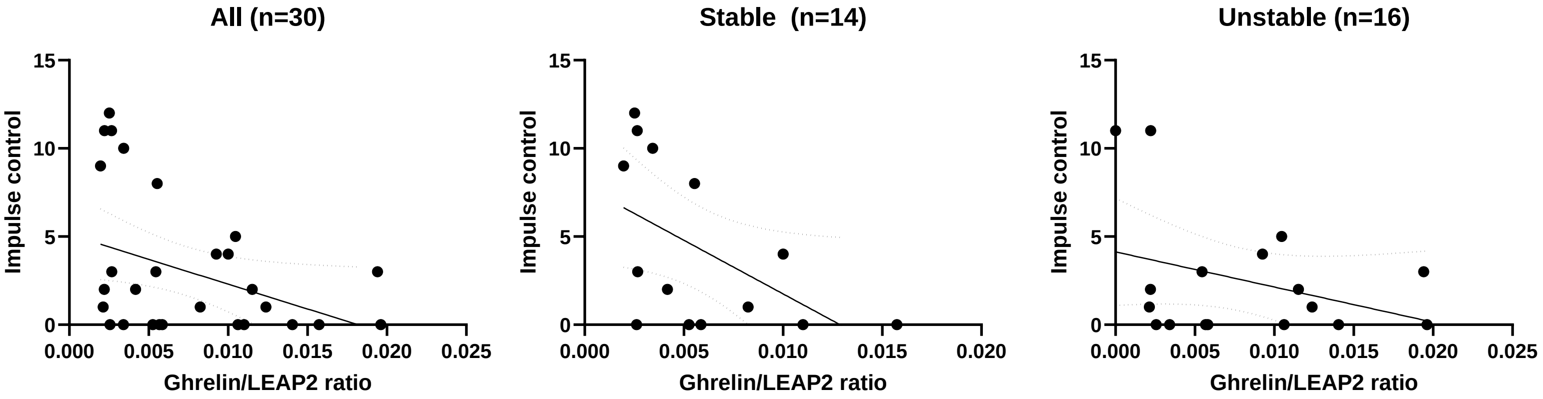

All (n=30)
Stable (n=14)
Unstable (n=16)
Impulse control
Ghrelin/LEAP2 ratio
0
5
10
15
0.000
0.005
0.010
0.015
0.020
0.025

Fig. S1

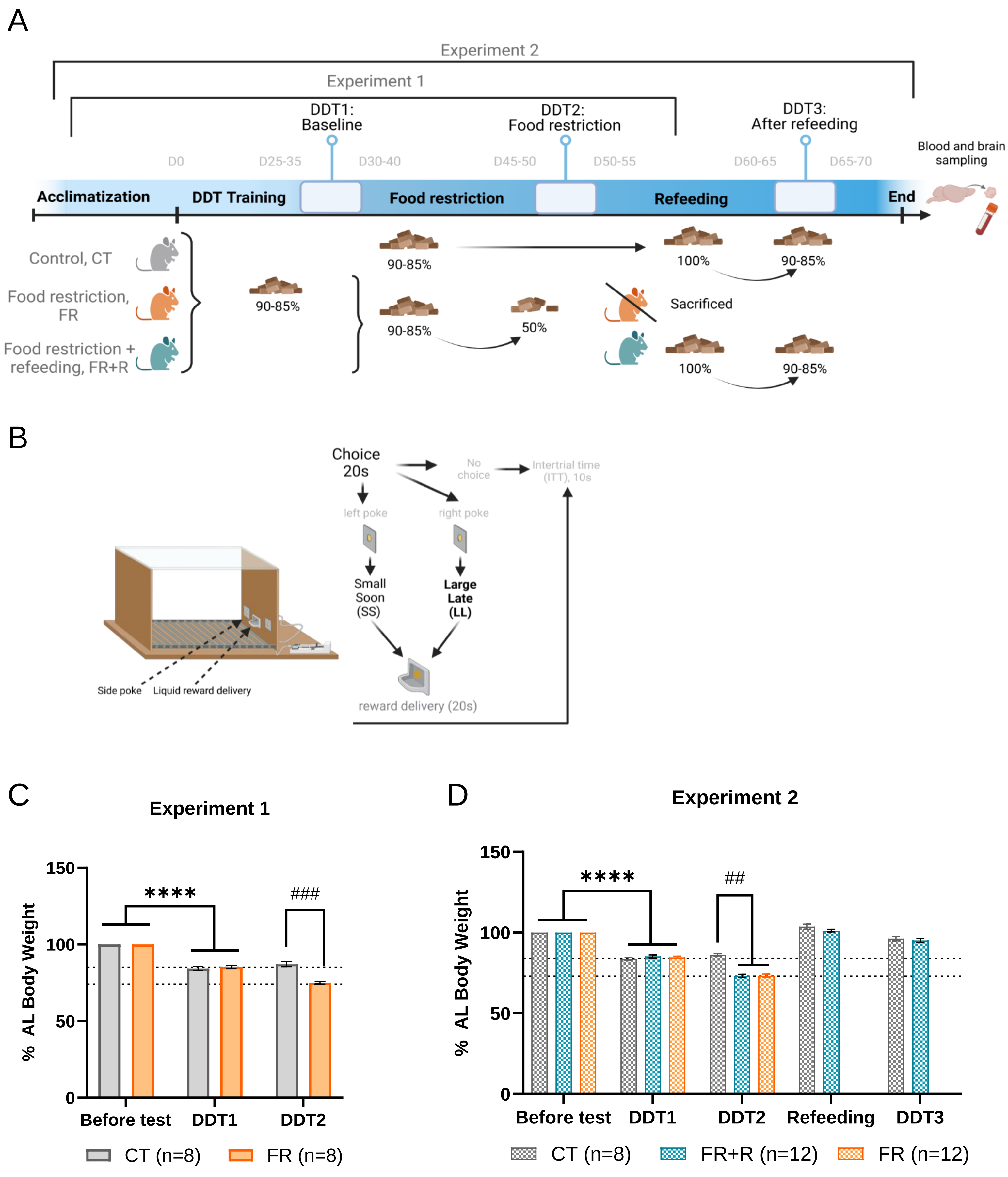
A
Experiment 2
Experiment 1
DDT1:
Baseline
DDT2:
Food restriction
DDT3:
After refeeding
D0
D25-35
D30-40
D45-50
D50-55
D60-65
D65-70
Blood and brain sampling
Acclimatization
DDT Training
Food restriction
Refeeding
End
Control, CT
Food restriction, FR
Food restriction + refeeding, FR+R
90-85%
90-85%
100%
90-85%
90-85%
50%
Sacrificed
100%
90-85%
B
Choice 20s
No choice
Intertrial time (ITT), 10s
left poke
right poke
Small Soon (SS)
Large Late (LL)
reward delivery (20s)
Side poke
Liquid reward delivery
C
Experiment 1
% AL Body Weight
150
100
50
0
****
###
Before test
DDT1
DDT2
CT (n=8)
FR (n=8)
D
Experiment 2
% AL Body Weight
150
100
50
0
****
##
Before test
DDT1
DDT2
Refeeding
DDT3
CT (n=8)
FR+R (n=12)
FR (n=12)

Fig. S2

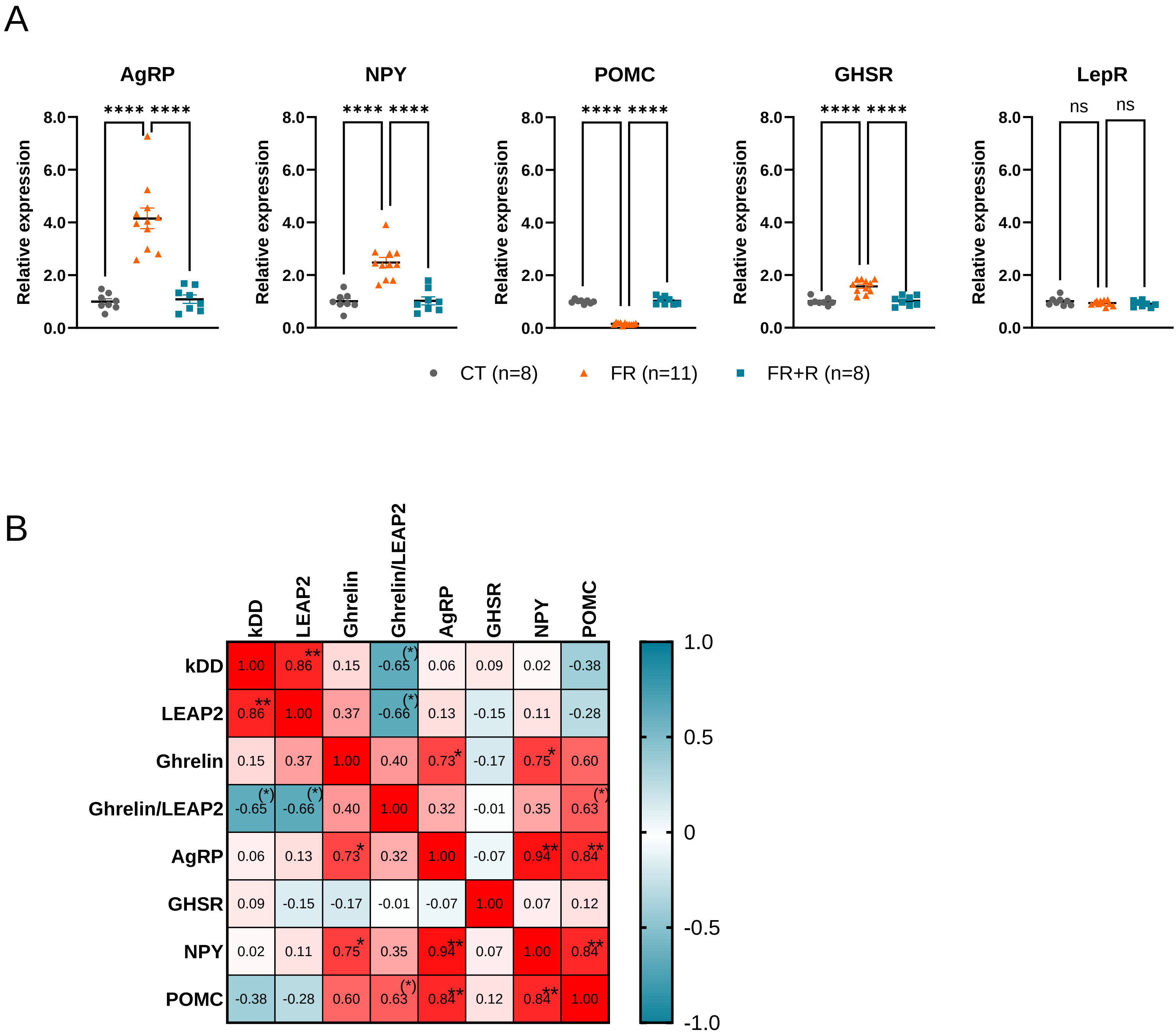
A
AgRP
NPY
POMC
GHSR
LepR
Relative expression
8.0
6.0
4.0
2.0
0.0
**** ****
ns
ns
CT (n=8)
FR (n=11)
FR+R (n=8)
B
kDD
LEAP2
Ghrelin
Ghrelin/LEAP2
AgRP
GHSR
NPY
POMC
kDD 1.00 0.86** 0.15 -0.65(*) 0.06 0.09 0.02 -0.38
LEAP2 0.86** 1.00 0.37 -0.66(*) 0.13 -0.15 0.11 -0.28
Ghrelin 0.15 0.37 1.00 0.40 0.73* -0.17 0.75* 0.60
Ghrelin/LEAP2 -0.65(*) -0.66(*) 0.40 1.00 0.32 -0.01 0.35 0.63(*)
AgRP 0.06 0.13 0.73* 0.32 1.00 -0.07 0.94** 0.84**
GHSR 0.09 -0.15 -0.17 -0.01 -0.07 1.00 0.07 0.12
NPY 0.02 0.11 0.75* 0.35 0.94** 0.07 1.00 0.84**
POMC -0.38 -0.28 0.60 0.63(*) 0.84** 0.12 0.84** 1.00
1.0
0.5
0
-0.5
-1.0

Fig. S3

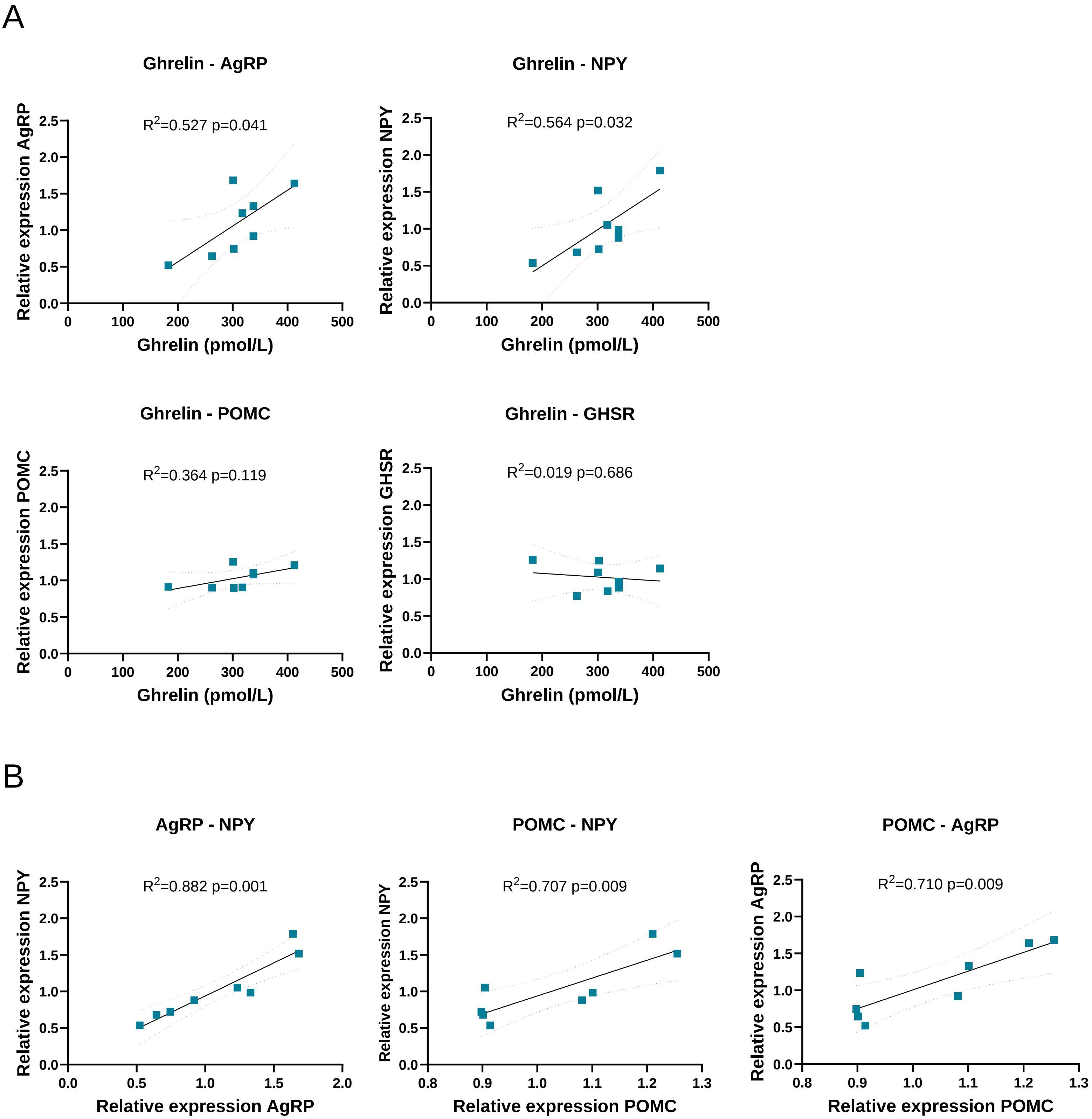
A
Ghrelin - AgRP
R2=0.527 p=0.041
Relative expression AgRP
Ghrelin (pmol/L)
Ghrelin - NPY
R2=0.564 p=0.032
Relative expression NPY
Ghrelin (pmol/L)
Ghrelin - POMC
R2=0.364 p=0.119
Relative expression POMC
Ghrelin (pmol/L)
Ghrelin - GHSR
R2=0.019 p=0.686
Relative expression GHSR
Ghrelin (pmol/L)
B
AgRP - NPY
R2=0.882 p=0.001
Relative expression NPY
Relative expression AgRP
POMC - NPY
R2=0.707 p=0.009
Relative expression NPY
Relative expression POMC
POMC - AgRP
R2=0.710 p=0.009
Relative expression AgRP
Relative expression POMC

Fig. S4

Food Restricted + Refed (n=7-8)

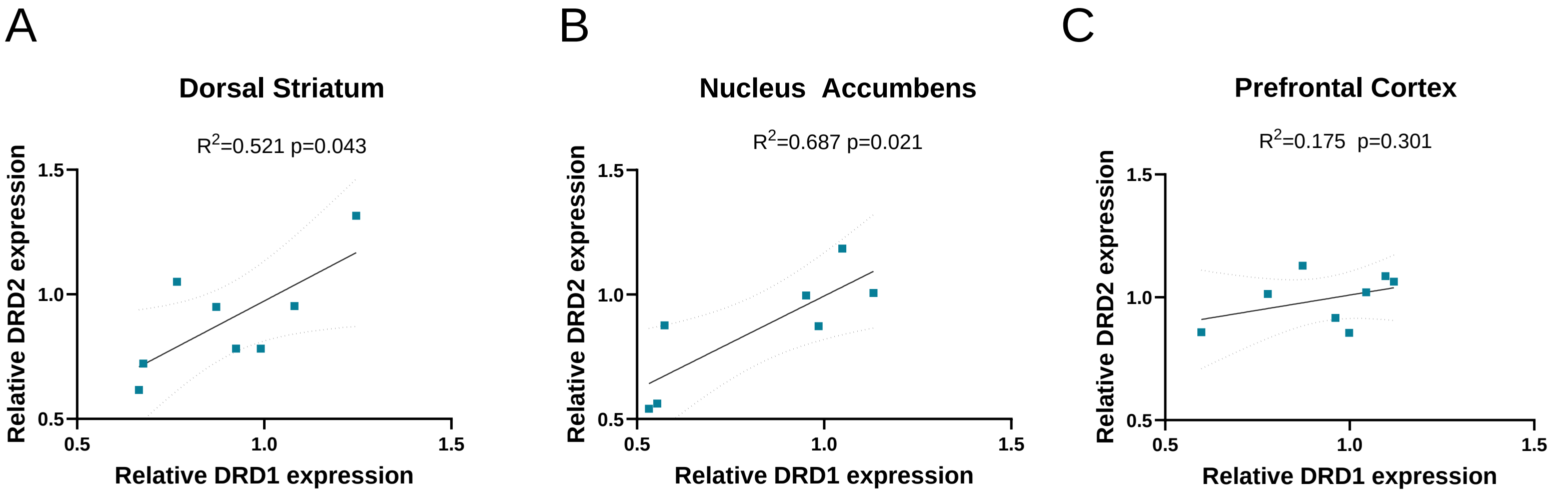

Fig. S5
